# Association of the *MMP12* rs652438 Variant with Lung Cancer Susceptibility: A Smoking-Stratified Analysis

**DOI:** 10.3390/biomedicines14102349

**Published:** 2026-10-15

**Authors:** Te-Chun Shen, Guan-Liang Chen, Liang-Wen Hang, Wen-Shin Chang, Shou-Cheng Wang, Ding-Han Chen, Nivetha Jeyabal, Yi-Chen Ke, Te-Chun Hsia, Da-Tian Bau, Chia-Wen Tsai

**Affiliations:** 1 Division of Pulmonary and Critical Care Medicine, Department of Internal Medicine, China Medical University Hospital, Taichung 404327, Taiwan; 2 School of Medicine, College of Medicine, China Medical University, Taichung 404333, Taiwan; 3 Division of Critical Care Medicine, Chu Shang Show Chwan Hospital, Nantou 55782, Taiwan; 4 National Defense Medical University, Taipei 114201, Taiwan; 5 Taichung Armed Forces General Hospital, Taichung 411228, Taiwan; 6 Graduate Institute of Biomedical Sciences, China Medical University, Taichung 404333, Taiwan; 7 Terry Fox Cancer Research Laboratory, Department of Medical Research, China Medical University Hospital, Taichung 404327, Taiwan; 8 Institute of Translational Medicine and New Drug Development, China Medical University, Taichung 404333, Taiwan; 9 Office of Research and Development, Asia University, Taichung 413305, Taiwan

**Keywords:** genotype, lung cancer, *MMP12*, polymorphism, smoking

## Abstract

**Background:** Matrix metalloproteinase 12 (MMP12) participates in extracellular-matrix remodeling and smoking-related pulmonary inflammation. This study evaluated the associations of *MMP12* rs2276109 and rs652438 with lung cancer susceptibility in a Taiwanese population and examined rs652438 according to smoking status. **Materials and Methods:** This case–control study included 358 patients with histologically confirmed lung cancer and 716 age- and sex-matched cancer-free controls. Genotypes were determined using polymerase chain reaction–restriction fragment length polymorphism analysis. Odds ratios (ORs) and 95% confidence intervals (CIs) were estimated under genotype, dominant, recessive, and allele models. Smoking-stratified estimates were adjusted for age and sex. **Results:** rs2276109 was not associated with lung cancer susceptibility. For rs652438, the CC genotype was associated with higher odds of lung cancer than the TT genotype (OR = 4.68, 95% CI = 2.00–10.99, *p* = 0.0003). Associations were also observed under the recessive (OR = 4.41, 95% CI = 1.89–10.32) and dominant (OR = 1.51, 95% CI = 1.12–2.03) models. The C allele was associated with higher lung cancer odds (OR = 1.62, 95% CI = 1.25–2.11). Among ever-smokers, the CC genotype remained associated with lung cancer after adjustment for age and sex (adjusted OR = 5.63, 95% CI = 2.17–15.38), whereas no significant association was observed among non-smokers. **Conclusions:** *MMP12* rs652438, but not rs2276109, was associated with lung cancer susceptibility in this Taiwanese population. The association was more apparent among ever-smokers, although formal genotype-smoking interaction analyses were not statistically significant. Independent replication and functional investigation are warranted.

## 1. Introduction

Lung cancer remains the most frequently diagnosed malignancy and the leading cause of cancer-related death worldwide. In 2024, approximately 2.6 million new lung cancer cases and 1.9 million lung cancer-related deaths were estimated globally [1]. Lung carcinogenesis is influenced by environmental and behavioral exposures, particularly cigarette smoking, as well as inherited genetic susceptibility [2]. Cigarette smoking is the predominant preventable risk factor and accounts for approximately 80–90% of lung cancer-related deaths [3,4]. Nevertheless, only a proportion of individuals exposed to tobacco smoke develop lung cancer, suggesting that inherited genetic variation may contribute to interindividual differences in susceptibility. Environmental carcinogenesis in lung cancer involves complex interactions between external exposures, inflammatory responses, oxidative injury, and molecular alterations that influence individual susceptibility [5].

Matrix metalloproteinases (MMPs) are a family of zinc-dependent proteases that regulate extracellular matrix (ECM) turnover and participate in inflammatory and tissue-remodeling processes [6]. MMP12, also known as macrophage elastase, is produced predominantly by macrophages and has broad proteolytic activity against ECM components. Experimental and clinical evidence indicates that MMP12 contributes to airway inflammation, ECM remodeling, and structural lung injury [7,8]. Within the pulmonary microenvironment, macrophage-derived MMP12 may influence both inflammatory responses and tissue remodeling [9,10]. These biological activities provide a plausible link between MMP12, smoking-related pulmonary injury, and lung carcinogenesis.

The human *MMP12* gene is located on chromosome 11q22.2 and comprises 10 exons and 9 introns [11]. Among its reported variants, rs2276109 and rs652438 have attracted particular attention because of their potential functional significance. The rs2276109 variant is located in the promoter region and may influence *MMP12* transcription by altering activator protein-1 binding and promoter activity [12,13]. In contrast, rs652438 is a nonsynonymous coding variant that results in an asparagine-to-serine substitution within the hemopexin-like domain, a region involved in substrate recognition and proteolytic activity [14,15]. These variants may therefore influence MMP12 expression or function and modify individual susceptibility to lung cancer.

**Figure 1:**
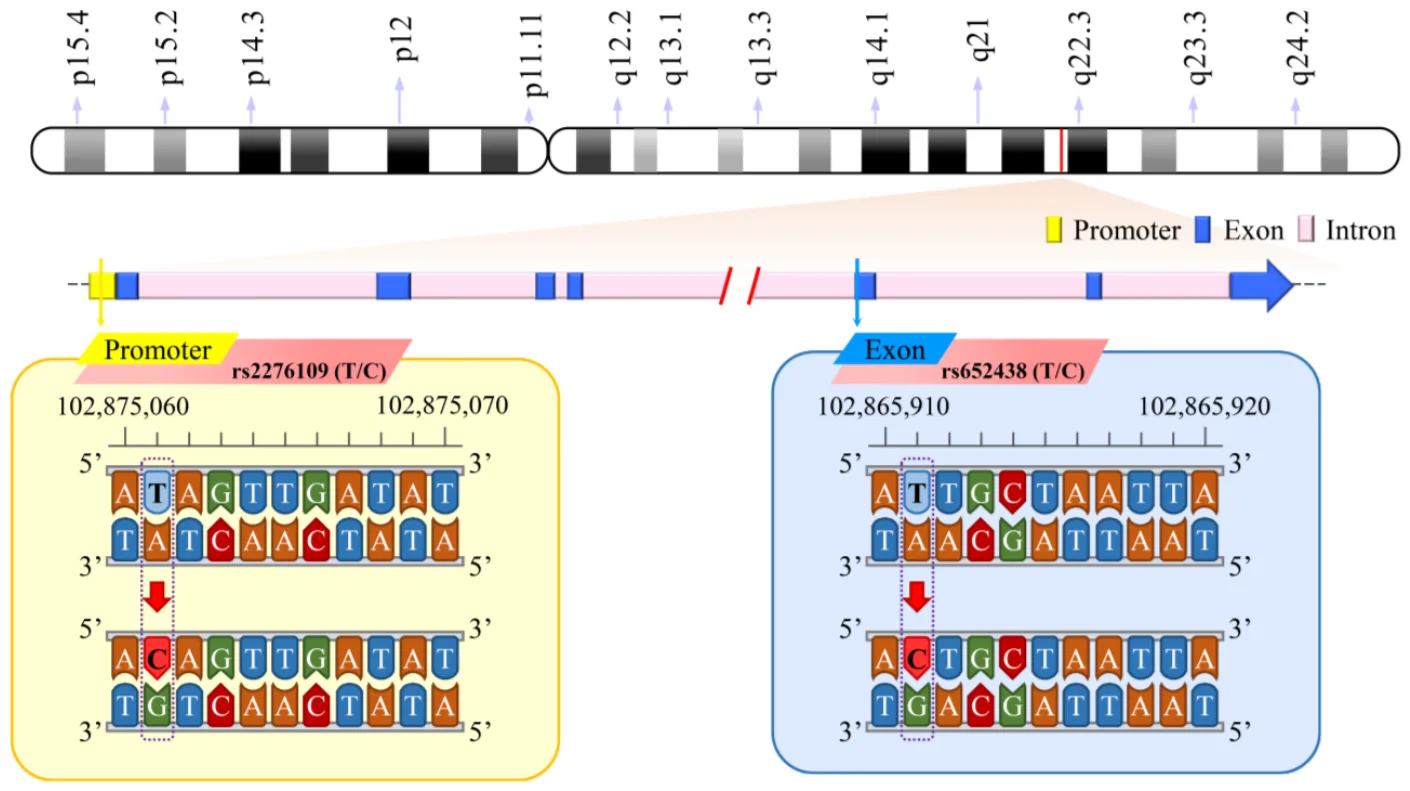
Genomic locations and sequence contexts of *MMP12* rs2276109 and rs652438. The *MMP12* gene is located on chromosome 11q22.2. The rs2276109 variant is located in the promoter region, whereas rs652438 is located in an exon. The enlarged panels show the genomic positions and local nucleotide sequences of the two T/C variants. Promoter, exon, and intron regions are shown in yellow, blue, and pink, respectively.

Previous studies have evaluated *MMP12* rs2276109 and rs652438 in relation to lung cancer susceptibility, progression, and prognosis, but their findings have been inconsistent across populations [12,16,17]. Evidence from East Asian populations remains limited, and the possible influence of smoking status on these genetic associations has not been fully clarified. Therefore, this case–control study investigated the associations of *MMP12* rs2276109 and rs652438 (Figure 1) with lung cancer susceptibility in a Taiwanese population. The association between rs652438 and lung cancer susceptibility was also evaluated separately among ever-smokers and non-smokers.

## 2. Materials and Methods

### 2.1. Study Population

A total of 358 consecutive patients with histologically confirmed lung cancer were enrolled at China Medical University Hospital according to previously established recruitment procedures [18]. Histological classification and pathological grading were independently confirmed by experienced pathologists.

The control group comprised 716 cancer-free individuals selected from a 15,000 database of healthy participants whose biospecimens were provided by the Health Examination Center. Controls were frequency-matched to patients with lung cancer by age and sex, and roughly smoking status. The final analytic dataset consisted of 358 patients with lung cancer and 716 cancer-free controls, thereby retaining the prespecified 2:1 control-to-case ratio. Peripheral venous blood samples (3–5 mL) were collected from all participants for genotyping with the assistance of the Tissue Bank and Health Examination Center.

Smoking status was obtained by self-report and was not independently verified using biochemical markers. Ever-smokers were defined as participants who reported a history of smoking and included both current and former smokers. Participants who reported never having smoked were classified as non-smokers.

The study protocol was approved by the Institutional Review Board of China Medical University Hospital (DMR100-IRB-284), and written informed consent was obtained from all participants. The demographic and clinical characteristics of the 358 patients with lung cancer and 716 matched controls are summarized in Table 1.

### 2.2. DNA Extraction and Storage

Genomic DNA was extracted from peripheral blood within 24 h of collection using the QIAamp Blood Mini Kit (Qiagen, Valencia, CA, USA) according to previously established protocols [19]. Purified genomic DNA was stored at −80 °C until use. Before genotyping, DNA samples from all participants were diluted to the required concentration, aliquoted into 96-well microplates, and stored at −20 °C as working stocks.

### 2.3. Genotyping of MMP12 rs2276109 and rs652438

The *MMP12* rs2276109 and rs652438 variants were genotyped using polymerase chain reaction–restriction fragment length polymorphism (PCR-RFLP) analysis, as previously described [20]. Genotype calls were independently evaluated by two trained investigators who were blinded to case–control status. Concordance between the two investigators was 100%. For rs652438, allele designations in the present study are reported according to the complementary strand used in the PCR-RFLP assay. Therefore, the T/C notation used here corresponds to the complementary allele orientation reported in some previous studies and databases.

### 2.4. Statistical Analysis

Continuous variables are presented as the mean and standard deviation and were compared between patients with lung cancer and controls using the unpaired Student’s *t*-test. Categorical variables were compared using Pearson’s chi-square test. Yates’ continuity correction was applied to 2 × 2 contingency tables when appropriate, whereas Fisher’s exact test was used when expected cell counts were small.

The genotype distributions of *MMP12* rs2276109 and rs652438 among the controls were assessed for deviation from Hardy–Weinberg equilibrium using the chi-square goodness-of-fit test. Differences in genotypic and allelic distributions between patients with lung cancer and controls were evaluated using the chi-square test or Fisher’s exact test, as appropriate. To account for multiple comparisons across the two investigated *MMP12* genotypes, Bonferroni correction methodology was applied, resulting in a corrected significance threshold of *p* < 0.025 (0.05/2). Associations between the *MMP12* variants and lung cancer susceptibility were expressed as odds ratios (ORs) with corresponding 95% confidence intervals (CIs). Genotype-specific, dominant, and recessive inheritance models were evaluated. Genotype trends were assessed using the Cochran–Armitage trend test. Potential effect modification by smoking was formally evaluated using multivariable logistic regression models with lung cancer status as the dependent variable. The models included rs652438 genotype, smoking status, and genotype-smoking interaction terms, with adjustment for age and sex. Smoking status was categorized as non-smoker or ever-smoker. For the genotype-specific analysis, rs652438 was treated as a categorical variable, with the TT genotype as the reference category. The overall genotype-by-smoking interaction was evaluated using a likelihood-ratio test comparing models with and without the interaction terms. Additional interaction analyses were conducted under the dominant (CT + CC versus TT) and recessive (CC versus TT + CT) genetic models. A two-sided *P*_interaction_ < 0.05 was considered statistically significant.

The strength of associations between the polymorphism and lung cancer risk was estimated using odds ratios (ORs) with corresponding 95% confidence intervals (CIs). All statistical tests were two-sided, and *p* < 0.05 was considered statistically significant.

## 3. Results

### 3.1. Participant Characteristics

The demographic and clinical characteristics of the 358 patients with lung cancer and 716 age- and sex-matched cancer-free controls are summarized in Table 1. The mean age was 64.0 ± 6.9 years among patients with lung cancer and 64.8 ± 6.8 years among controls, with no statistically significant difference between the groups (*p* = 0.0708). The distributions of sex (*p* = 0.3642) and smoking status (*p* = 0.2282) were also comparable between the groups. Among the patients with lung cancer, adenocarcinoma was the most common histological subtype, accounting for 60.9% (218/358) of cases, followed by squamous cell carcinoma (29.6%, 106/358). The remaining 9.5% (34/358) had other histological subtypes.

### 3.2. Association of MMP12 Genotypes with Lung Cancer Susceptibility

The genotype distributions of *MMP12* rs2276109 and rs652438 among the 358 patients with lung cancer and 716 cancer-free controls are presented in Table 2. The genotype distributions of both variants among the controls conformed to Hardy–Weinberg equilibrium (rs2276109, *p* = 0.3396; rs652438, *p* = 0.9877).

For rs2276109, neither the heterozygous CT genotype nor the homozygous CC genotype was significantly associated with lung cancer susceptibility compared with the TT reference genotype [CT: odds ratio (OR) = 1.07, 95% confidence interval (CI) = 0.66–1.74, *p* = 0.8814; CC: OR = 3.03, 95% CI = 0.50–18.23, *p* = 0.3399]. No significant association was observed under either the recessive model (CC vs. TT + CT: OR = 3.01, 95% CI = 0.50–18.14, *p* = 0.3405) or the dominant model (CT + CC vs. TT: OR = 1.14, 95% CI = 0.72–1.83, *p* = 0.6568). The genotype trend was also not statistically significant (*p* = 0.4143).

For rs652438, the CT genotype was not significantly associated with lung cancer susceptibility compared with the TT reference genotype (OR = 1.32, 95% CI = 0.97–1.81, *p* = 0.0936). In contrast, the CC genotype was significantly associated with higher odds of lung cancer (OR = 4.68, 95% CI = 2.00–10.99, *p* = 0.0003). Significant associations were also observed under the recessive model (CC vs. TT + CT: OR = 4.41, 95% CI = 1.89–10.32, *p* = 0.0005) and the dominant model (CT + CC vs. TT: OR = 1.51, 95% CI = 1.12–2.03, *p* = 0.0077). The genotype trend was statistically significant (*p* = 0.0004). After Bonferroni correction, the associations of rs652438 with lung cancer susceptibility remained statistically significant for the CC genotype (*p* = 0.0006), recessive model (*p* = 0.0010), dominant model (*p* = 0.0154), and genotype trend (*p* = 0.0008).

### 3.3. Association of MMP12 Alleles with Lung Cancer Susceptibility

The allele distributions of *MMP12* rs2276109 and rs652438 are presented in Table 3. The rs2276109 C allele was not significantly associated with lung cancer susceptibility compared with the T allele (OR = 1.21, 95% CI = 0.78–1.88, *p* = 0.4646). The association of the rs652438 C allele with lung cancer susceptibility also remained significant after Bonferroni correction (*p* = 0.0006).

In contrast, the rs652438 C allele was more frequent among patients with lung cancer than among controls (16.1% vs. 10.5%) and was significantly associated with higher odds of lung cancer compared with the T allele (OR = 1.62, 95% CI = 1.25–2.11, *p* = 0.0003). This allele-based result was consistent with the genotype associations presented in Table 2.

### 3.4. Smoking-Stratified Analysis of MMP12 rs652438

The association between *MMP12* rs652438 and lung cancer susceptibility was examined according to smoking status (Table 4). Among ever-smokers, the CC genotype was significantly associated with higher odds of lung cancer compared with the TT genotype (OR = 6.41, 95% CI = 2.30–17.87, *p* = 0.0001), and the association remained significant after adjustment for age and sex (aOR = 5.63, 95% CI = 2.17–15.38). Significant associations were also observed among ever-smokers under the dominant (OR = 1.57, 95% CI = 1.13–2.19, *p* = 0.0089) and recessive (OR = 6.02, 95% CI = 2.17–16.74, *p* = 0.0002) models. These associations remained significant after Bonferroni correction. In contrast, no significant genotype association was observed among non-smokers.

Formal genotype-smoking interaction analyses were additionally performed for *MMP12* rs652438. The overall interaction was not statistically significant when rs652438 was analyzed as a categorical genotype variable (*P*_interaction_ = 0.438). Similarly, no significant interaction was observed under the dominant (*P*_interaction_ = 0.601) or recessive (*P*_interaction_ = 0.209) genetic models. Therefore, the stronger association observed among ever-smokers should be interpreted as a smoking-stratified association rather than definitive evidence of a statistically significant genotype-smoking interaction.

## 4. Discussion

The present case–control study evaluated the associations of two potentially functional *MMP12* variants, rs2276109 and rs652438, with lung cancer susceptibility in a Taiwanese population. The principal finding was that rs652438, but not rs2276109, was associated with lung cancer susceptibility. Individuals carrying the rs652438 CC genotype had higher odds of lung cancer than those carrying the TT genotype, with consistent associations observed under the recessive and dominant models. The rs652438 C allele was also more frequent among patients with lung cancer than among controls. In the smoking-stratified analysis, the association with the CC genotype was more apparent among ever-smokers and remained significant after adjustment for age and sex. However, formal genotype-smoking interaction analyses did not demonstrate statistically significant interactions for the categorical genotype, dominant, or recessive models (*P*_interaction_ = 0.438, 0.601, and 0.209, respectively). Therefore, the stronger association observed among ever-smokers should be interpreted as a smoking-stratified association rather than definitive evidence of effect modification by smoking. All the above associations were checked carefully since our cohort is of relatively moderate, and rather modest differences in allelic or genotypic frequencies, and as a matter of fact, the association of rs652438 remained statistically significant after Bonferroni correction (Table 2, Table 3 and Table 4). In addition, the present study should be interpreted as a focused candidate-variant investigation rather than a genome-wide discovery study. Its relatively modest sample size and limited SNP coverage restrict the generalizability of the findings, and independent validation in substantially larger populations is necessary.

Previous studies of *MMP12* rs652438 have produced heterogeneous findings. Su and his colleagues found no overall association with lung cancer susceptibility in a predominantly Caucasian population, although subgroup-specific associations were reported [16]. Similarly, Sun and his colleagues found no significant individual association between rs652438 and lung cancer susceptibility in a Chinese population [12]. In contrast, Heist and his colleagues examined survival among patients with stage I non-small cell lung cancer rather than cancer susceptibility and therefore addressed a different clinical endpoint [17]. Differences in ethnicity, genetic background, linkage disequilibrium structure, environmental exposure, study design, phenotype definition, and statistical power may partly account for the inconsistent observations across studies. Accordingly, the present findings should be interpreted as population-specific evidence requiring independent replication rather than as confirmation of a previously established association.

Differences in genetic background, linkage disequilibrium patterns, environmental exposures, study design, and sample size may contribute to the heterogeneous findings across populations. As mentioned in Materials and Methods Section 2.3, the allele nomenclature for rs652438 also differs among reports because the present study used the complementary strand, consequently, the T/C labels used here correspond to the complementary alleles reported in some previous studies. Among the controls in the present study, the rs652438 T-allele frequency was 89.5%, which was similar to the frequency reported for East Asian populations in the NCBI dbSNP database (89.94%) [https://www.ncbi.nlm.nih.gov/snp/rs652438 (accessed on 15 Sep 2026)]. This similarity supports the plausibility of the observed allele distribution but does not establish population representativeness or exclude selection bias. Independent replication in other Taiwanese and East Asian populations remains necessary.

The different findings for rs2276109 and rs652438 may also be biologically relevant. rs2276109 is located in the promoter region and may influence *MMP12* transcription through altered activator protein-1 binding, whereas rs652438 is a nonsynonymous coding variant that produces an asparagine-to-serine substitution within the hemopexin-like domain [12,13,14,15]. Because this domain contributes to substrate recognition and proteolytic activity, rs652438 could plausibly influence MMP12 function. Nevertheless, the present study evaluated germline genotypes rather than MMP12 expression or enzymatic activity. The observed association therefore cannot establish that rs652438 directly alters MMP12 function or identify the biological mechanism underlying the association.

The stronger association observed among ever-smokers is biologically plausible because MMP12 is closely involved in macrophage-mediated inflammation and tissue remodeling following cigarette-smoke exposure. Human studies have reported an association between active smoking and higher circulating MMP12 concentrations [21]. Experimental studies have also shown that cigarette-smoke exposure increases pulmonary macrophage infiltration and MMP12 expression and that these inflammatory changes may persist after smoking cessation [22]. These observations are compatible with the possibility that smoking-related pulmonary inflammation may influence the phenotypic consequences of *MMP12* variation. However, the present data provide only indirect support for this interpretation. Smoking status was self-reported and analyzed dichotomously, with current and former smokers combined as ever-smokers. Information regarding smoking duration, intensity, pack-years, and time since cessation was unavailable. Moreover, the absence of a statistically significant association among non-smokers does not demonstrate that the magnitude of the association differs significantly between smoking strata.

Lung cancer comprises biologically heterogeneous histological subtypes. Although adenocarcinoma and squamous cell carcinoma accounted for 60.9% and 29.6% of cases, respectively, subtype-specific genetic analyses were not performed because further stratification of the uncommon rs652438 CC genotype would have resulted in unstable estimates (Table 1). Clinical and experimental evidence have further indicated that the role of MMP12 in lung cancer is context dependent. Increased tumor-associated MMP12 expression has been linked to recurrence, metastasis, and poorer outcomes in some studies [23]. In contrast, experimental evidence suggests that stromal MMP12 may inhibit tumor angiogenesis through the generation of angiostatin [24]. Circulating MMP12 concentrations have also varied across clinical studies [25,26]. More broadly, altered expression of other molecular biomarkers has also been associated with the biological behavior and clinical outcomes of lung cancer. For example, Xiao et al. reported that increased mPRα/PAQR7 expression was associated with malignant phenotypes and poorer overall survival in lung squamous cell carcinoma [27]. These findings emphasize that germline variation, tumor expression, stromal expression, and circulating protein concentrations represent distinct biological measures and should not be interpreted interchangeably. The present study addresses inherited susceptibility and does not determine the effects of rs652438 on tumor behavior, circulating MMP12 concentrations, treatment response, or prognosis.

Several strengths of this study should be noted. All patients had histologically confirmed lung cancer, controls were matched to cases by age and sex at a 2:1 ratio, and genotype calls were independently evaluated by two investigators blinded to case–control status, with 100% concordance. The genotypic distributions among the controls also conformed to Hardy–Weinberg equilibrium. In addition, the association of rs652438 was observed across genotype-specific, allele, dominant, recessive, and trend analyses, providing internal consistency.

This study also has several limitations. First, its single-center case–control design is susceptible to selection bias and cannot establish causality. Second, the rs652438 CC genotype was uncommon, particularly after stratification by smoking status. Therefore, the corresponding ORs were estimated from small cell counts and had wide confidence intervals, indicating substantial statistical imprecision. The magnitude of these ORs should consequently be interpreted cautiously. Third, smoking exposure was based on self-report and may therefore have been subject to recall or classification error. Also, smoking exposure was not quantified using pack-years or other measures of cumulative exposure. Current and former smokers could not be analyzed separately, and quantitative smoking information, including cigarettes per day, smoking duration, cumulative pack-years, and time since cessation, was unavailable. Consequently, dose–response relationships and residual confounding related to cumulative tobacco exposure could not be evaluated. Fourth, the proportion of ever-smokers among controls was relatively high (78.6%), because controls were recruited from an institutional health-examination cohort rather than through population-based sampling. During control selection, smoking status was approximately frequency-matched to that of the case group. Therefore, the observed smoking prevalence in controls should not be interpreted as representative of the general Taiwanese population. Controls were recruited from individuals undergoing health examinations at the same institution rather than through population-based sampling. Such individuals may differ from the source population in health-related behaviors and healthcare utilization, and selection bias therefore cannot be excluded. Fifth, although the association appeared stronger among ever-smokers, the formal genotype-smoking interaction tests were not statistically significant. Therefore, the smoking-stratified findings should not be interpreted as definitive evidence of effect modification by smoking. The relatively small number of rs652438 CC carriers may also have limited the statistical power of the interaction analyses. Finally, the study included neither functional experiments nor an independent validation cohort. Larger studies incorporating detailed smoking-exposure data, functional characterization, and replication in independent populations are warranted to further evaluate potential genotype-smoking interactions.

## 5. Conclusions

In this Taiwanese case–control study, *MMP12* rs652438 was associated with higher odds of lung cancer, whereas rs2276109 was not. The association with the rs652438 CC genotype was more apparent among ever-smokers; however, this stratified finding does not establish a genotype-by-smoking interaction. Larger independent studies with more detailed smoking-exposure information are needed to clarify the observed association.

## Figures and Tables

**Table 1:** Demographic and clinical characteristics of 358 patients with lung cancer and 716 age- and sex-matched cancer-free controls.

Characteristics	Controls *n* = 716 *n*	%	Mean (SD)	Patients *n* = 358 *n*	%	Mean (SD)	*p*-Value
Age, years			64.8 (6.8)			64.0 (6.9)	0.0708 ^a^
Sex							
Male	488	68.1		254	70.9		
Female	228	31.9		104	29.1		0.3642 ^b^
Smoking status							
Ever-smokers	563	78.6		293	81.8		
Non-smokers	153	21.4		65	18.2		0.2282 ^b^
Histological subtype							
Adenocarcinoma				218	60.9		
Squamous cell carcinoma				106	29.6		
Other				34	9.5		

Data are presented as n (%) or mean (SD), as appropriate. SD, standard deviation. ^a^ Unpaired Student’s *t*-test. ^b^ Chi-square test with Yates’ continuity correction.

**Table 2:** Associations of *MMP12* rs2276109 and rs652438 genotypes with lung cancer susceptibility.

Genotype/Model	Patients Total *n* = 358 *n* (%)	Controls Total *n* = 716 *n* (%)	OR (95% CI)	aOR (95% CI) ^a^	*p*-Value ^b^	BA *p*-Value ^c^
**rs2276109**						
TT	328 (91.6)	663 (92.6)	1.00 (Reference)	1.00 (Reference)		
CT	27 (7.6)	51 (7.1)	1.07 (0.66–1.74)	1.10 (0.68–1.76)	0.8814	
CC	3 (0.8)	2 (0.3)	3.03 (0.50–18.23)	2.87 (0.49–16.48)	0.3399	
*p* for trend					0.4143	
**Recessive model**						
TT + CT	355 (99.2)	714 (99.7)	1.00 (Reference)	1.00 (Reference)		
CC	3 (0.8)	2 (0.3)	3.01 (0.50–18.14)	2.65 (0.49–15.86)	0.3405	
**Dominant model**						
TT	328 (91.6)	663 (92.6)	1.00 (Reference)	1.00 (Reference)		
CT + CC	30 (8.4)	53 (7.4)	1.14 (0.72–1.83)	1.12 (0.70–1.77)	0.6568	
**rs652438**						
TT	260 (72.6)	573 (80.0)	1.00 (Reference)	1.00 (Reference)		
CT	81 (22.6)	135 (18.9)	1.32 (0.97–1.81)	1.28 (0.93–1.76)	0.0936	0.1872
CC	17 (4.8)	8 (1.1)	**4.68 (2.00–10.99)**	**4.13 (1.88–9.45)**	**0.0003**	**0.0006**
*p* for trend					**0.0004**	**0.0008**
**Recessive model**						
TT + CT	341 (95.2)	708 (98.9)	1.00 (Reference)	1.00 (Reference)		
CC	17 (4.8)	8 (1.1)	**4.41 (1.89–10.32)**	**3.99 (1.83–8.94)**	**0.0005**	**0.0010**
**Dominant model**						
TT	260 (72.6)	573 (80.0)	1.00 (Reference)	1.00 (Reference)		
CT + CC	98 (27.4)	143 (20.0)	**1.51 (1.12–2.03)**	**1.47 (1.11–1.84)**	**0.0077**	**0.0154**

OR, odds ratio; CI, confidence interval; HWE, Hardy–Weinberg equilibrium. ^a^ Adjusted for age, sex, and smoking status using multivariable logistic regression; ^b^ Pearson’s chi-square test with Yates’ continuity correction or Fisher’s exact test, as appropriate. Genotype trends were evaluated using the Cochran–Armitage trend test. ^c^ *p*-values calculated based on Bonferroni-adjusted analysis. The genotype distributions among the controls conformed to HWE for both rs2276109 (*p* = 0.3396) and rs652438 (*p* = 0.9877). Statistically significant values are shown in bold.

**Table 3:** Associations of *MMP12* rs2276109 and rs652438 alleles with lung cancer susceptibility.

Variant/Allele	Patients Total *n* = 716 *n* (%)	Controls Total *n* = 1432 *n* (%)	OR (95% CI)	*p*-Value ^a^	BA *p*-Value ^b^
**rs2276109**					
T	683 (95.4)	1377 (96.2)	1.00 (Reference)		
C	33 (4.6)	55 (3.8)	1.21 (0.78–1.88)	0.4646	
**rs652438**					
T	601 (83.9)	1281 (89.5)	1.00 (Reference)		
C	115 (16.1)	151 (10.5)	**1.62 (1.25–2.11)**	**0.0003**	**0.0006**

OR, odds ratio; CI, confidence interval. ^a^ Chi-square test with Yates’ continuity correction. ^b^ *p*-values calculated based on Bonferroni-adjusted analysis. Statistically significant values are shown in bold.

**Table 4:** Associations of *MMP12* rs652438 genotypes with lung cancer susceptibility stratified by smoking status.

Smoking Status	Genotype	Controls, *n*	Patients, *n*	OR (95% CI)	aOR (95% CI) ^a^	*p*-Value ^b^	BA *p*-Value ^c^	*P* for Interaction
**Non-smokers**	TT	120	48	1.00 (reference)	1.00 (Reference)			
	CT	30	15	1.25 (0.62–2.53)	1.31 (0.64–2.38)	0.6616		
	CC	3	2	1.67 (0.27–10.29)	1.59 (0.33–9.64)	0.6274		
**Ever-smokers**	TT	453	212	1.00 (reference)	1.00 (Reference)			
	CT	105	66	1.34 (0.95–1.90)	1.29 (0.89–1.86)	0.1160	0.2320	
	CC	5	15	**6.41 (2.30–17.87)**	**5.63 (2.17–15.38)**	**0.0001**	**0.0002**	0.438 ^1^
**Dominant model**								
Non-smokers	CT + CC vs. TT	33/120	17/48	1.29 (0.66–2.53)	1.15 (0.59–2.42)	0.5751		
Ever-smokers	CT + CC vs. TT	110/453	81/212	**1.57 (1.13–2.19)**	**1.65 (1.19–2.35)**	**0.0089**	**0.0178**	0.601 ^2^
**Recessive model**								
Non-smokers	CC vs. TT + CT	3/150	2/63	1.59 (0.26–9.73)	1.62 (0.35–9.61)	0.6359		
Ever-smokers	CC vs. TT + CT	5/558	15/278	**6.02 (2.17–16.74)**	**5.67 (2.11–14.68)**	**0.0002**	**0.0004**	0.209 ^3^

OR, odds ratio; aOR, adjusted odds ratio; CI, confidence interval. ^a^ Adjusted for age and sex using multivariable logistic regression. ^b^ Chi-square test with Yates’ continuity correction or Fisher’s exact test, as appropriate. ^c^ *p*-values calculated based on Bonferroni-adjusted analysis. Statistically significant values are shown in bold. ^1^ Overall genotype × smoking interaction based on a 2-degree-of-freedom likelihood-ratio test comparing models with and without the genotype-by-smoking interaction terms; ^2^ Interaction under the dominant genetic model; ^3^ Interaction under the recessive genetic model.

## Data Availability

The original contributions presented in this study are included in the article. Further inquiries can be directed to the corresponding authors.
